# Lacosamide intake during pregnancy increases the incidence of foetal malformations and symptoms associated with schizophrenia in the offspring of mice

**DOI:** 10.1038/s41598-020-64626-9

**Published:** 2020-05-06

**Authors:** Beatriz López-Escobar, Rut Fernández-Torres, Viviana Vargas-López, Mercedes Villar-Navarro, Tatyana Rybkina, Eloy Rivas-Infante, Ayleen Hernández-Viñas, Concepción Álvarez del Vayo, José Caro-Vega, José A. Sánchez-Alcázar, Antonio González-Meneses, M. Ángel Carrión, Patricia Ybot-González

**Affiliations:** 1 Grupo de Neurodesarrollo, Hospital Universitario Virgen del Rocio/Instituto de Biomedicina de Sevilla (IBIS)/CSIC/Universidad de Sevilla, Sevilla, 41013 Spain; 2 Departamento de Química Analítica, Facultad Química, Universidad Sevilla, Sevilla, Spain; 3 Centro de Investigación en Salud y Medio Ambiente (CYSMA), Universidad de Huelva, Huelva, Spain; 4 Departamento de Fisiología, Anatomía y Biología Celular, Universidad Pablo de Olavide, 41013 Sevilla, Spain; 5 Behavioral Neurophysiology Laboratory, School of Medicine, Universidad Nacional de Colombia, Bogotá, Colombia; 6 Unidad de Gestión Clínica de Anatomía Patología, Hospital Universitario Virgen del Rocío, Sevilla, Spain; 7 Unidad de Gestión Clínica de Farmacia, Hospital Universitario Virgen del Rocío and Universidad de Sevilla, Sevilla, Spain; 8 Centro Andaluz de Biología del Desarrollo (CABD), and Centro de Investigación Biomédica en Red: Enfermedades Raras, Instituto de Salud Carlos III, CSIC, Universidad Pablo de Olavide, 41013 Sevilla, Spain; 9 Unidad de Gestión Clínica de Pediatría, Hospital Universitario Virgen del Rocío and Universidad de Sevilla, Sevilla, Spain; 10 Unidad de Gestión Clínica de Neurología y Neurofisiología, Hospital Universitario Virgen Macarena, Sevilla, 41009 Spain

**Keywords:** Developmental biology, Neuroscience

## Abstract

The use of first and second generation antiepileptic drugs during pregnancy doubles the risk of major congenital malformations and other teratogenic defects. Lacosamide (LCM) is a third-generation antiepileptic drug that interacts with collapsing response mediator protein 2, a protein that has been associated with neurodevelopmental diseases like schizophrenia. The aim of this study was to test the potential teratogenic effects of LCM on developing embryos and its effects on behavioural/histological alterations in adult mice. We administered LCM to pregnant mice, assessing its presence, and that of related compounds, in the mothers’ serum and in embryonic tissues using liquid chromatography coupled to quadrupole/time of flight mass spectrometry detection. Embryo morphology was evaluated, and immunohistochemistry was performed on adult offspring. Behavioural studies were carried out during the first two postnatal weeks and on adult mice. We found a high incidence of embryonic lethality and malformations in mice exposed to LCM during embryonic development. Neonatal mice born to dams treated with LCM during gestation displayed clear psychomotor delay and behavioural and morphological alterations in the prefrontal cortex, hippocampus and amygdala that were associated with behaviours associated with schizophrenia spectrum disorders in adulthood. We conclude that LCM and its metabolites may have teratogenic effects on the developing embryos, reflected in embryonic lethality and malformations, as well as behavioural and histological alterations in adult mice that resemble those presented by patients with schizophrenia.

## Introduction

Epilepsy is a common chronic illness suffered by 65 million people worldwide with a global annual incidence of 50 cases per 100,000 and a prevalence of 700 per 100,000 inhabitants^1^. Antiepileptic drugs (AEDs) are the first line of treatment for epilepsy and some of them are also prescribed for other conditions, like chronic migraine, pain syndromes and psychiatric disorders^2^. Traditional AEDs (first- and second-generation) are often effective, although their utility may be limited in 30% of patients who develop chronic epilepsy, patients who are often refractory to these AEDs, and who therefore continue to experience recurrent seizures^3^. For these patients, the most appropriate treatment is either a combination of AEDs or the use of novel (third-generation) AEDs.

Pregnancy represents a unique therapeutic problem due to the existence of two individuals: the mother or patient and the foetus that may or may not suffer from the disease. As such, while the mother may benefit from a given pharmacological treatment, this may have an adverse effect on the foetus. The use of AEDs in women of childbearing age represents a clinical challenge, particularly due to the 2- to 4-fold risk of inducing a congenital malformation and adverse cognitive outcomes during pregnancy^4^. In the past 10 years, promising third-generation AEDs have been introduced that may be used in women of childbearing age, although their real risk of teratogenicity and of producing foetal malformation remains unknown.

The use of mice as animal models has provided researchers the opportunity to gain insight into how specific treatments might affect neurodevelopmental and behavioural disorders^5^. This approach is particularly important for the teratogenic studies of a given drug for a chronic disease. Lacosamide (LCM) is a third-generation AED for which no teratogenic effects have been described in mammals. LCM (R-2-acetamido-N-benzyl-3- methoxypropionamide) is known commercially as Vimpat, and it is an amino functional molecule with anticonvulsant properties but with an ill-defined mechanism of action. LCM selectively modulates the voltage-gated sodium channels (VGSC), and the collapsing response mediator protein 2 (CRMP2)^6^. It is also an effective anticonvulsant in animal models, clinically approved as an adjunct therapy to reduce seizure frequency in patients with uncontrolled, partial onset seizures^1^. The weak protein binding of LCM (<15%) favours its use with other AEDs, as it does not affect cytochrome P-450 or produce strong side-effects. Neurologists recognize that its versatility might favour its use in women of childbearing age. Little is known about the teratogenic effects of LCM or its impact on neurodevelopment^7,8^, and given that approximately 40% of pregnancies worldwide are unintended^9^, we have assessed how treating pregnant mice with LCM (in equivalent doses to those used to treat epilepsy in humans) affects embryonic development, as well as the behaviour of the neonatal and young adult offspring. As LCM modulates the activity of neurodevelopmentally-related proteins like CRMP2 and/or VGSCs^10,11^, LCM might potentially induce neuropsychiatric disorders indirectly. Accordingly, genetic linkage studies on humans and behavioural studies on mice have associated the CRMP2 protein (also called DPYSL2) with abnormal behaviour related to the schizophrenia spectrum and other psychiatric disorders^12–17^. Our data indicate that LCM administration to pregnant mice may not only have teratogenic effects, but also, it may have neurodevelopmental consequences inducing complex behaviours changes associated with schizophrenia spectrum disorders in the offspring of mice exposed to LCM when they reach adulthood.

## Results

### Effect of LCM on embryo development

**Figure 1:**
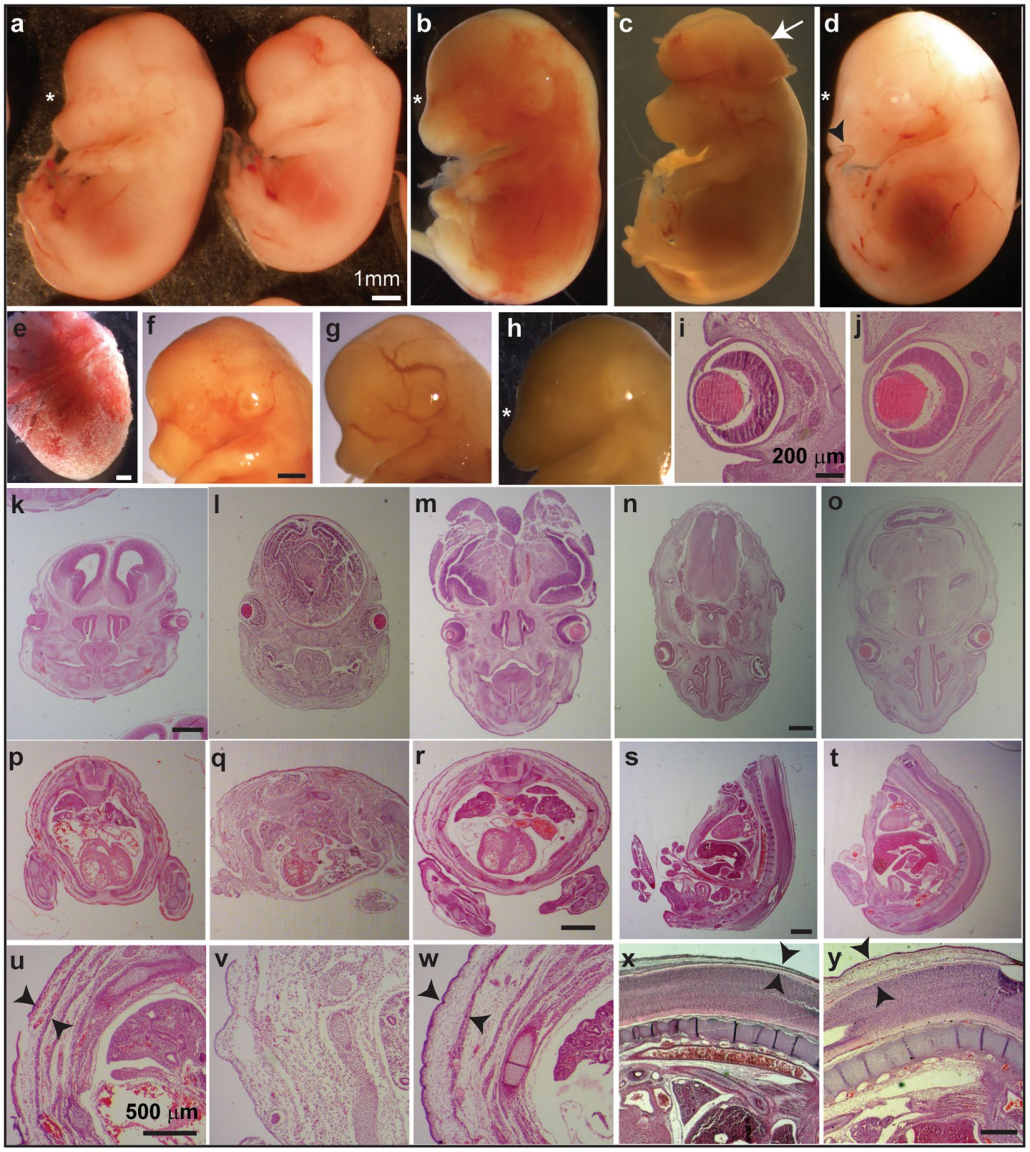
Teratogenic effect of Lacosamide in early embryo development. (**a–d**) E14.5 embryos and corresponding H&E stained sections (**i–y**). (**e–h**) diverse macroscopic malformations: calk-like decidua (**e**); abnormal circulatory system **(f,g**); abnormal cranial shape (**h**). (**k**–**m**) coronal sections through the head of (**a**), (control left), (**b**,**c**), respectively. (**i,j,n,o**) transverse sections through the head of a control embryo (not shown) and (**d**) respectively. (**p**–**r**) transverse sections of the torso of (**a–c**) at the heart level. (**s–t**) sagittal sections of the body at heart level of a control embryo (not shown) and (**d**), respectively. (**u–y**) magnification of the sections of the dorsal skin of control in (**a–c**), control (not shown) and (**d**), respectively. The histological studies from the embryos from the low dose group (10 mg/kg) indicate: one embryo that developed generalized oedema (**d**) in the subcutaneous tissue and in the soft cranial tissues, mainly at the back of the eye (**i,j,n,o,s,t,x,y**); one embryo with exencephaly and subcutaneous oedema (**c,r,w**), with a caudo-rostral deterioration leading to stronger damage to the caudal cranial neural tissue (**m**); and one dead embryo without evidence of a lethal malformation (**b**), yet displaying considerable necrosis (**l,q,v**). Two more embryos in this group (40 mg/kg) were found inside the decidua covered in a calk-like layer (**e**), one of which was necrotic while the second appeared normal. in (**c**) indicates exencephaly; in (**d**) points to the curled end of the tail. * in (**a**) indicates a normal cranial shape and in (**b,d,h**), an altered cranial shape. in (**u**,**w**,**x**,**y**) marks the thickness of the dorsal skin. Scale bars: 1 mm in **(a**) (also in **b**–**d**), (**e**,**f**) (also for **g**,**h**), (**k**) (also for **l**,**m**), (**n**) (also for **o**), (**r**) (also for **p**,**q**), (**s**) (also for **t**); 200 μm in (**i)** (also for **j**); 500 μm in (**u**) (also for **v**,**w**), and in (**x**) (also for **y**).

We first explored the effects of LCM on embryo development, administering the dams different daily doses of LCM (10, 40, 80 or 120 mg/kg, n = 10, 5, 5, and 7, respectively). When analysed on gestation day (E)14.5, there was a clear decrease in the number of embryos/litter as the doses of LCM administered to the dams increased, accompanied by an increase in the number of resorptions (see Table 1): controls, 14 ± 0.4 embryos/litter; low doses of LCM (treated with 10 or 40 mg/kg LCM), 12.2 ± 0.1 embryos/litter; and high doses of LCM (80 or 120 mg/kg LCM) 9.5 ± 0.4 embryo/litter, (*p* = 0.02). Other parameters of the embryos were also considered, comparing the embryos isolated from dams that received high or low doses of LCM to the controls. As such, there was a clear increase in the number of developmental malformations as the dose of LCM augmented (see Table 1), including oedemas, exencephaly, alteration of cranial shape and the circulatory system (Fig. 1). As organogenesis is completed in the embryo by E14.5, from this point onwards only changes in size are likely to be affected by the administration of LCM. Indeed, the crown-rump length of embryos was shorter in the embryos isolated from dams that received higher doses of LCM (see Table 1).

**Table 1:** Effect of Lacosamide on viable litters, resorptions, litter size and fetal abnormalities.

LCM mg/kg	Dams (no.)	Lost Litter (no.)	Resorpt (%)	Emb (no.)	Emb/dam	CR length (cm)	Malformation (%)
**Control**^**a**^	**5**	**0**	**0**	**70**	**14** ± **0.4**	**1.11** ± **0.09**	**1.4**
**Low doses**^**a**^	**15**	**0**	**3.2**	**184**	**12.2** ± **0.1**	**1.1** ± **0.1**	**5.4**
10	10	0	4	117	11.7	1.18	4.3
40	5	0	1.5	67	13.4	1.09	7.4
**High doses**^**a**^	**12**	**2**	**1.75**	**114**	**9.5** ± **0.4**^**b**^	**1.06** ± **0.08**^**b**^	**6.1**
80	5	1	0	41	8.2	1.03	12.2
120	7	1	2.5	73	10.4	1.08	2.7

^a^The data represent the means ± S.E.M. unless otherwise indicated.

^b^Statistically different from no-drug, pair-fed group, p < 0.05: Resorpt, resorptions; Emb, embryos.CR: Crown-rump.

### Effect of maternal LCM administration on early postnatal development

**Figure 2:**
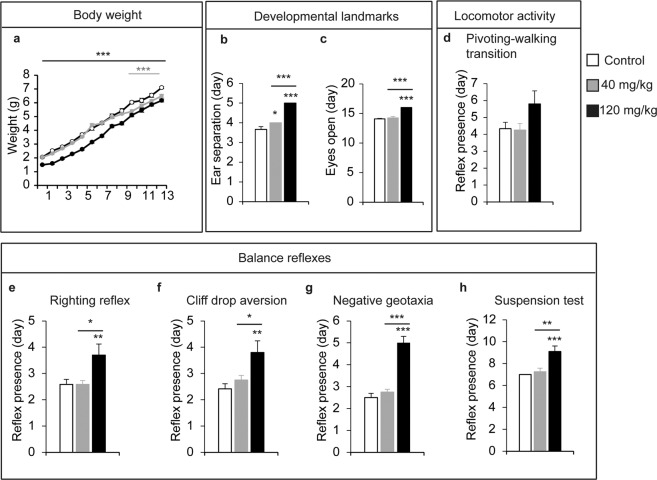
Lacosamide administration during gestation affects postnatal body weight and neurobehavioral landmarks in a dose-dependent manner. (**a**) The time course of body weight evolution of pups at birth to postnatal day 13 was quantified. (**b**–**h**) postanatal day of presence of the development marks, locomotor activity and balance reflexes measured in the Fox Battery tests. Development landmarks (**b**, ear separation and **c**, eye opening), locomotor activity (**d**, pivoting-walking transition) and balance reflexes (**e**–**h**, righting, cliff drop aversion, negative geotaxia and suspension tests). In all the case mean ± SEM are represented. N were 12, 12 and 10 for vehicle, 40 and 120 mg/kg of Lacosamide injected mice respectively. *p < 0.05; **p < 0.01; and ***p < 0.001.

After analysing the effects of LCM on early embryo development, we studied the behaviour of those animals prenatally exposed to LCM, only studying these pups born to dams that were administered 40 mg/kg (*lower dose*) or 120 mg/kg (*higher dose*) LCM. To increase the number of animals obtained and to avoid early embryo loss, dams were administered daily systemic injections of 40 or 120 mg/kg of LCM from the second day of gestation to birth, a regime that produced a similar rate of litter loss as the original administration regime. With this protocol, 1/5 dams that received 40 mg/kg and 2/8 that received 120 mg/kg never gained weight, suggesting that they lost their litters, which did not occur with any of the control dams (n = 4). Indeed, some neonatal lethality was detected among the pups from LCM treated dams but not among the control pups (n = 27): 3/33 (9%) at the lower dose; and 10/65 (15.38%) at the higher dose. Moreover, the pups from dams exposed to a higher LCM dose weighed less (1.4 g ± 0.04/pup) than those born to dams that received the lower dose of LCM (2.04 g ± 0.027/pup) or the control pups (2.05 g ± 0.06/pup) when weighed on the second day after delivery (P2: Fig. 2a). Further, the entire litter (11 pups) from a dam exposed to higher dose died in the perinatal nursing period (P15-16), after gaining weight very slowly (from P2 to P13: 2 g) relative to other pups under the same condition (6.1 g).

### Systemic administration of LCM during gestation provokes a dose-dependent delay in early postnatal development

**Figure 3:**
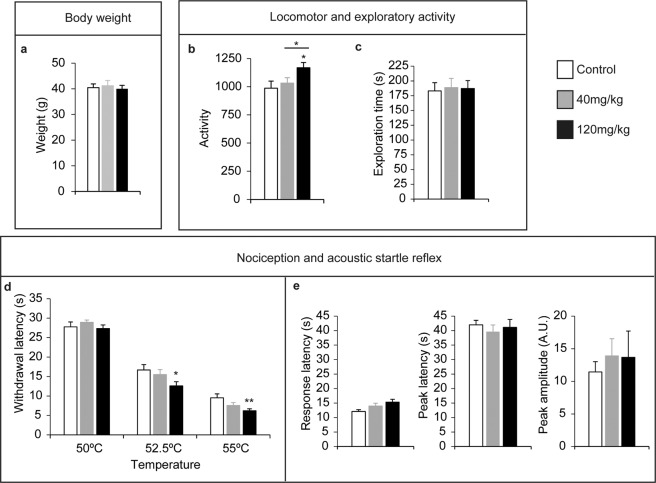
Administration of Lacosamide during gestation produced mild effects on locomotion and sensory gait in adulthood. To test the global health of adult male mice born from control dams or different doses of Lacosamide during their gestation, their body weight, locomotor, exploratory and sensorial activities were evaluated. (**a**) The adult body weight of mice. (**b**,**c**) Total locomotor and novel exploration activities were measured in an open field for 5 minutes. (**d**) Thermal nociception was evaluated with a hot plate test at 50, 52.5 and 55 °C. (**e**) Acoustic startle response and peak latencies, and maximum peak amplitude were measured in the acoustic startle reflex. Error bars represent the SEM. *p < 0.05; and **p < 0.01. N = at least ten animals were analysed for each condition and protocol.

To determine the effect of maternal LCM administration on the postnatal psychomotor development of mice, we performed the behavioural tests included in Fox’s battery from P1 to P13. Postnatal somatometric development and reflexological analyses showed LCM produced a dose-dependent delay in body weight (2-way ANOVA for treatment-age interaction: *F*_(132,11)_ = 2.73, *p* = 0.015: Fig. 2a), in developmental landmarks (ear separation: *F*_(31,2)_ = 59.71, *p* < 0.001; and eye opening: *F*_(30,2)_ = 36.27, *p* < 0.001: Fig. 2b,c), in locomotor activity (pivoting-walking transition: (*F*_(31,2)_ = 2.69, *p* = 0.083: Fig. 2d), and in balance reflexes (righting reflex: *F*_(31,2)_ = 5.7, *p* = 0.007; cliff drop aversion: *F*_(31,2)_ = 6.43, *p* = 0.004; negative geotaxia: *F*_(31,2)_ = 40.84, *p* < 0.001, Fig. 2e–g), or the suspension test (*F*_(31,2)_ = 11.64, *p* < 0.001: Fig. 2h). Interestingly, low dose LCM administration only caused mild effects on body weight and ear separation relative to the control mice. Moreover, the body weight differences observed postnatally disappeared in the adults for both LCM doses (*F*_(49.2)_ = 0.13, *p* = 0.86: Fig. 3a). Together, these data indicate that administration of high LCM dose during embryonic development causes postnatal delays, a mark of neurodevelopmental diseases.

### LCM administration during gestation affects locomotor and thermal perception but not exploratory activity or acoustic startle in adult mice

To determine whether LCM administration during gestation affects behaviour in adulthood, locomotor and exploratory activity we also evaluated 3–5 month old mice in the open field test. Mice born to dams that received high-dose LCM displayed enhanced locomotor activity (*F*_(49,2)_ = 3.45, *p* = 0.04) but similar novel objects exploration as control mice (Fig. 3b,c). We also evaluated the effect of LCM administration during gestation on nociception and on the acoustic startle reflex of adult mice (Fig. 3d,e), and only mice born to dams administered 120 mg/kg LCM during gestation displayed significant hyperalgesia in the hot plate test at 52 and 55 degrees relative to control mice (2-way ANOVA for LCM *F*_(147,8)_ = 5.37, *p* = 0.0056: Fig. 3d). Conversely, LCM administration during gestation did not modify any parameters of the acoustic startle reflex, such as the peak latencies and maximum peak response (Fig. 3e).

### LCM administration during gestation alters mood, learning and memory, and induces symptoms associated with neuropsychiatric diseases in adult mice

**Figure 4:**
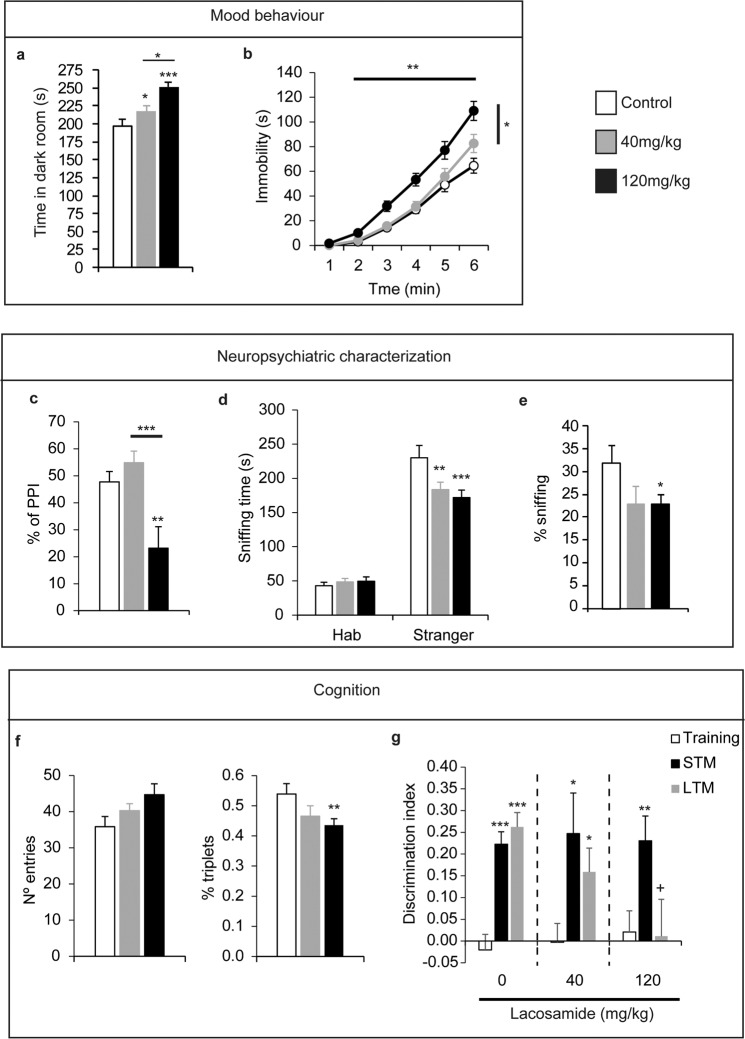
Lacosamide administration during gestation induced abnormal behaviour in adult male mice. (**a,b**) Mood behaviour in adult male mice born to dams administrated different doses of Lacosamide was assessed in the emergence (**a**) and tail suspension tests (**b**). In the emergence test, the time in a dark box was measured over 5 minutes. In the suspension test, the accumulated time spent immobile (s) over 5 minutes in the tail suspension test was evaluated. (**c–e**) Neuropsychiatric characterization was performed by pre-pulse inhibition (PPI) of acoustic startle (**c**, percentage of inhibition), sociability (**d**, sniffing time measured in seconds) and fear to a predator (**e**, sniffing time on material used by rats) tests. (**f,g**) Cognitive functioning was tested working memory in the Y maze (**f**), and the novelty preference (discrimination index) in object recognition memory task (**g**). In object recognition memory test (*) represents a significant difference between each memory tests (STM and LTM) with respect to training session in the same pharmacological group; and (+) represents a significant difference between the distinct pharmacological groups in the same behavioural session. The error bars represent the SEM. N were 16, 17 and 19 for vehicle, 40 and 120 mg/kg of Lacosamide injected mice respectively. *p < 0.05; **p < 0.01; and ***p < 0.001.

To study mood-related behaviour in adult mice exposed to LCM during gestation, 3–5-month-old mice were subjected to the emergence test and the tail suspension test to assess anxiety- and depressive-related behaviours, respectively (Fig. 4a,b). The emergence test revealed a LCM dose-dependent increase in time spent in the dark compartment, an anxiogenic-related behaviour (control 197.08 s ± 10.14, 40 mg/kg 217.2 ± 11.19, and 120 mg/kg *in utero* LCM 251 s ± 5.25 s; *F*_(49,2)_ = 8.67, *p* < 0.001: Fig. 4a). Moreover, mice born to dams that received 120 mg/kg of LCM during gestation displayed a significant increase in immobility, depressive-related signal, when compared to the controls (control 64.5 ± 6.01, 40 mg/kg 82.58 ± 7.38, and 120 mg/kg *in utero* LCM 109.0 ± 7.69 s; *F*_(49,2)_ = 9.06, *p* < 0.001: Fig. 4b).

To model the possible neuropsychiatric symptoms caused by LCM administration during gestation, we evaluated pre-pulse inhibition (PPI), a phenomenon by which a weak acoustic pre-stimulus suppresses the response to an acoustic startle stimulus. While the amplitude of the acoustic startle response was comparable between control mice and those exposed to LCM (Fig. 3e), the PPI of the acoustic startle response was significantly reduced in those mice from dams exposed to 120 mg/kg LCM (control 47.7 ± 3.88, 40 mg/kg 54.85 ± 4.27, and 120 mg/kg LCM 23.19 ± 7.9%; *F*_(49,2)_ = 9.3, *p* < 0.001: Fig. 4c). We then evaluated sociability and panic behaviours. In the sociability test, mice exposed to LCM *in utero* displayed a weaker social interaction with a strange mouse (control 230.17 ± 17.86, 40 mg/kg 183.4 ± 11.01, and 120 mg/kg *in utero* LCM 172.0 ± 10.78 s; *F*_(49,2)_ = 5.18; *p* = 0.014: Fig. 4d). Finally, fear of a predator was evaluated by exposure to material used by rats, a natural predator of mice. In this test, both groups of mice exposed to LCM spent less time sniffing the predator´s material (control 32.04 ± 3.68, 40 mg/kg 22.85 ± 4.07, and 120 mg/kg *in utero* LCM 22.91 ± 2.08%; *F*_(49,2)_ = 3.94, *p* = 0.035: Fig. 4e), although the difference was only significant for the mice that were born to dams exposed to the high dose of LCM.

Cognitive deficiencies in adult mice treated *in utero* with LCM were assessed through several learning and memory tests. We evaluated arm alternation of the mice in the hippocampal-dependent Y-maze working memory test (Fig. 4f), revealing a LCM-dependent decrease in alternation (*F*_(49,2)_ = 3.699, *p* = 0.04) and a mild increase in locomotion (*F*_(49,2)_ = 2.70, *p* = 0.087) between the control mice and those from dams exposed to LCM. The object recognition memory test revealed that adult mice that were exposed to LCM developed dose-dependent impaired long-term memory (LTM), when evaluated 24 hours after the training session (the discrimination indices were: control 0.26 ± 0.03, 40 mg/kg 0.15 ± 0.054, and 120 mg/kg *in utero* LCM 0.01 ± 0.084; *F*_(49,2)_ = 4.26, *p* = 0.022: Fig. 4g). By contrast, no effects on short-term memory were observed 1 hour after the training session (*F*_(49,2)_ = 0.03, *p* = 0.96).

Globally, mice born to dams treated with a high dose of LCM during gestation exhibited a complex behavioural phenotype, including features of hyperactivity, depression, anxiety and fear; impaired sociability and LTM; and loss of PPI in the acoustic startle reflex. These alterations phenocopy the positive, negative, and cognitive symptoms in human patients suffering schizophrenia (revised by^18^).

### LCM administration during gestation alters different neocortical areas

**Figure 5:**
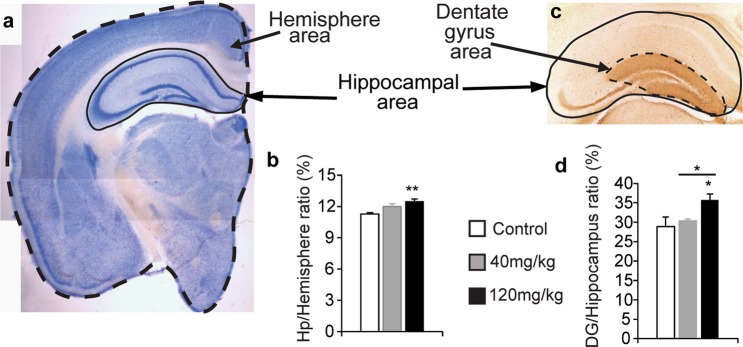
The relative size of the hippocampus increases in adult male mice born to dams treated with a high dose of Lacosamide (LCM). Relative hippocampal and dentate gyrus size were analysed in Nissl stained sections and those stained by immunohistochemistry for calbindin protein, respectively. (**a**) Nissl stained section in which the hemisphere and hippocampal area are labelled (defined in dashed and solid lines, respectively). (**b**) The relative hippocampus size in adult mice born to dams treated with the vehicle alone or the different doses of LCM. N = 8,8 and 11 mice for vehicle, 40 and 120 mg/kg of Lacosamide injected mice respectively. (**c**) Calbindin immunohistochemistry in a section in which the hippocampus and dentate gyrus area are labelled (defined in solid and dashed lines, respectively). (**d**) The relative dentate gyrus size in adult mice born to dams treated with the vehicle (control) and the different doses of LCM (N = 4, 4 and 6 respectively). Error bars represent the SEM. *p < 0.05; and **p < 0.01.

As LCM treatment during gestation provoked behavioural alterations when mice reached adulthood that were reminiscent of the changes associated with schizophrenia, we studied whether they also associated with morphological alterations in the adult mouse neocortex. Small but significant LCM dose-dependent alterations in the size of hippocampus relative to the brain, and of the dentate gyrus (DG) relative to the hippocampus, were detected in Nissl stained tissue and immunohistochemically labelled for calbindin: hippocampus: brain ratios - control 11.28 ± 0.13, 40 mg/kg 12 ± 0.25, and 120 mg/kg LCM 12.47 ± 0.26% (*F*_(22,2)_ = 5.27, *p* = 0.012: Fig. 5a,b); and DG:hippocampus ratios - control 28.9 ± 2.46, 40 mg/kg 30.38 ± 0.48, and 120 mg/kg LCM 35.67 ± 1.63% (*F*_(11,2)_ = 4.57; *p* = 0.035: Fig. 5c,d).

**Figure 6:**
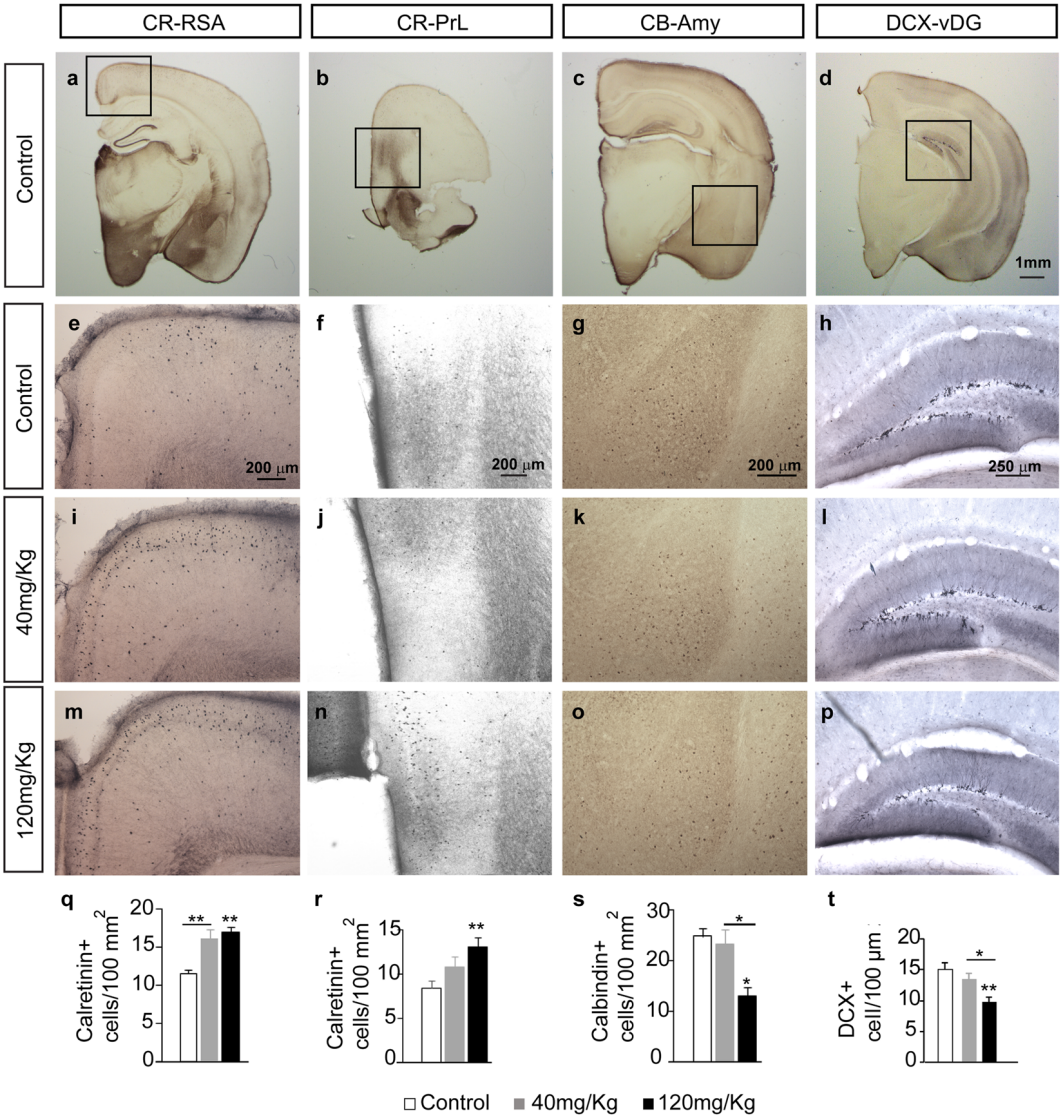
Adult male mice born to dams that received Lacosamide treatment during gestation presented morphological alterations in the neocortex. To detect morphological alterations in the neocortex of adult male mice born to dams treated with the vehicle alone or with different doses of LCM during gestation, calretinin (CR), calbindin (CB) and doublecortin (DCX) immunohistochemistry (IHC) was performed. The figure shows representative microphotographs and quantifications from several neocortex areas. (**a**) Neuronal density in the agranular retrosplenial (RSA) and (**b**), prelimbic (PrL) cortices were determined by IHC for CR. (**c**) Alterations in the amygdala were determined by IHC for CB. (**d**) The density of immature neurons in the dentate gyrus was quantified by DCX IHC. In each case, representative microphotographs (1–3) and quantifications are shown (4), where the error bars represent the SEM. *p < 0.05; and **p < 0.01. N = 9, 8 and 11 mice were processed for each primary antibody and pharmacological treatment. Scale bars: 1 mm in (**d**) (also for **a**–**c**); 200 μm in (**e**) (also for **i**,**m**), in (**f**) (also for **j**,**n**), and in (**g**) (also for **k**,**o**); 250 μm in (**h**) (also for **l**,**p**).

Impaired social behaviour is associated with morphological alterations to the prefrontal cortex, especially in the agranular retrosplenial (RSA) and prelimbic (PrL) cortices^19^ (Fig. 6a,b). Immunohistochemistry for the interneuron marker calretinin (CR) was used to assess these regions and interestingly, we observed a LCM dose-dependent increase in the number of CR neurons in both the RSA and PrL cortices: RSA (cells/100 µm² - control 11.55 ± 0.57, 40 mg/kg 16.18 ± 1.23, and 120 mg/kg LCM 16.94 ± 0.7; *F*_(28,2)_ = 11.16; *p* < 0.001: Fig. 6a,e,i,m,q); and PrL cortex (cells/100 µm² - control 8.45 ± 0.82, 40 mg/kg 10.83 ± 1.12 and 120 mg/kg LCM 13.03 ± 1.08; *F*_(28,2)_ = 5,33; *p* = 0.013: Fig. 6b,f,j,n,r). Fear is related to alterations in the amygdala^20^ and we detected a dose-dependent decrease in the number of calbindin (CB) labelled neurons in the amygdala (cells/100 µm^2^) in mice from dams exposed to LCM (control 24.88 ± 1.55, 40 mg/kg 23.28 ± 2.98, and 120 mg/kg LCM 13 ± 1.8; *F*_(28,2)_ = 10.54, *p* < 0.001: Fig. 6c,g,k,o,s). Finally, adult hippocampal neurogenesis in the hippocampus is related to mood, anxiety, and depression, as well as altered cognition, learning, and memory^21–23^. Doublecortin (DCX) is a known marker for immature adult neurons^24^, and a clear dose-dependent loss of DCX labelling (cells/100 µm²) was detected by immunohistochemistry, reflecting a decrease in adult hippocampal neurogenesis in ventral DG associated with LCM exposure (control 15.09 ± 1.02, 40 mg/kg 13.46 ± 1.03, and 120 mg/kg LCM 9.75 ± 0.85; *F*_(28,2)_ = 8,43, *p* = 0.002: Fig. 6d,h,l,p,t).

### LCM and related compounds (RC) in serum and embryo

We set out to confirm that the higher doses of LCM administered were associated with a higher concentration of this AED in the serum of the dams, and hence that their embryos were likely to be exposed to more LCM. However, no relationship was found between the dose of LCM administered and the concentration of this AED in the serum taken from 2 dams in each group 24 h post-injection, detecting concentrations ranging from 5–133 ng/ml, consistent with a previous report^25^. The effects observed in embryos born to dams administered LCM have not been reported previously in mammals or individuals that received LCM directly. Therefore, we assessed the status of the LCM and its metabolites in 30 embryos (22 placebo and 8 embryos from treated dams) chosen to reflect each dose administered, and taking into account the total LCM concentration in the serum. Thus, 4 embryos from each group (low dose and high dose) were analysed by high-resolution mass spectrometer (MS) and MS^e^ experiments (to identify LCM related metabolites and degradation compounds). Unaltered LCM and the two previously reported compounds RC1 and RC2^26^ were detected in the serum of all mothers and in a single embryo (from a mother given a low dose of LCM), which would suggest that these are generated as a first step in the metabolism of LCM. In terms of the metabolites, the presence of RC-6 and -7 appeared to be related to higher doses (≥40 mg/kg), while RC-1, RC-3 and RC-4 were more closely associated with lower doses (≤40 mg/kg). RC-2 and RC-5 seem not to be correlated with any dose administered to the dams. There also seemed to be no relationship between the dose administered and the number of related compounds found in the embryos. However, embryos from dams that received 120 mg/kg contained completely different metabolic pattern to the rest of embryos, presenting unique metabolites (RC-8, RC-9 and RC-10). The compounds found in the embryos were also studied in the mother’s serum and only RC-1 and RC-2 coincided in all mothers, whereas RC-4 was found only in the mother of the 2 embryos that contained this compound. RC-9 was present in the serum of all mothers, with the exception of one dam treated with 120 mg/kg of LCM and her embryos. Although the rest of the RCs detected in the embryos were not present in the mothers, intermediate compounds were found in some of the mothers but not in their embryos. Hence, certain metabolic processes appear to occur in the mother but not in the embryo, and vice versa (see Table 2 and the supplementary data for a summary of these studies and the metabolic routes involved). These differences in the metabolites found may explain the specific effects of this AED on the embryos.

**Table 2:** Presence of Lacosamide and its metabolites in embryos exposed to low dose (10 mg/kg and 40 mg/kg) or high dose (80 mg/kg and 120 mg/kg) of Lacosamide during gestation.

Embryos	Maternal Dose	LCM compound
Embryo1 and 2	10 mg/kg	RC 1
		RC 2
		RC 3
		RC 5
		LCM
Embryo 3 and 4	40 mg/kg	RC 3
		RC 4
		RC 5
		RC 6
		RC 7
Embryo 5 and 6	80 mg/kg	RC 2
		RC 5
		RC 6
		RC 7
Embryo7 and 8	120 mg/kg	RC 8
		RC 9
		RC 10

Abbreviations: LCM, Lacosamide; RC 1, C_7_H_9_N; RC 2, C_12_H_16_N_2_O_3_; RC 3, C_4_H_9_NO_2_; RC 4, C_9_H_10_O; RC 5, C_11_H_15_NaO_2_; RC 6, C_13_H_17_NO_3_; RC 7, C_13_H_17_NO_6_; RC 8, C_10_H_13_NO_2_; RC 9, C_11_H_18_N_2_O_2_; RC 10, C_13_H_18_N_2_O_5_.

## Discussion

Patients suffering from epilepsy usually require lifelong treatment with AEDs. LCM is a third-generation antiepileptic drug frequently used in epileptic patients, even during gestation. Here we found that LCM administration to mice during gestation affects embryonic development, clearly producing detrimental effects in mid-gestation, as well as in postnatal and adult male mice.

There has been much research into first- and second- generation AEDs and their teratogenic effects in mammals. When administrated during pregnancy, these drugs may cause neurobehavioral alterations in the adult offspring without other apparent malformations^27^, although little attention has been paid to these possible effects of LCM in mammals. Indeed, the lethality and teratogenic effects of LCM observed in our study corroborate data obtained in other species^7,8^. While some embryos exposed to LCM suffered morphological alterations or died during gestation, most of them were born without any clear malformations. Nevertheless, we detected an LCM dose-dependent delay in somatometric and reflexological responses in these mice during early postnatal development, suggesting that exposure to high LCM doses provokes some neurodevelopmental delay. However, although some of the somatometric parameters had reverted to a normal range by the time these mice reached adulthood (e.g., the body weight), mice born to dams treated with high doses of LCM exhibited a complex behavioural phenotype that included: hyperactivity (Fig. 3b,c), passiveness (Fig. 4b), increased defensiveness (Fig. 4a,e), reduced sociability (Fig. 4d), impaired long-term memory (Fig. 4f,g), altered somatosensorial processing and heat hyperalgesia (Fig, 3d and 3e), and importantly a loss of pre-pulse inhibition in the acoustic startle reflex (Fig. 4c). Globally, these behavioural alterations phenocopy the positive, negative and cognitive symptoms required for a diagnosis of schizophrenia in human^18^. From the perspective of the Research Domain Criteria (RDoC) launched by the NIMH, behavioural changes observed in the present study would be related to those phenotypes associated with schizophrenia spectrum disorders. In the six domains included in the RDoC system, the following was observed: (a) increased defensive response and reduced novelty seeking (negative valence); (b) reduced motivation to escape aversive situation (positive valence); (c) impaired memory (cognitive system); (d) reduced sociability (social processes); (e) hyperactivity and reduced inhibition (arousal and regulatory systems); and (f) hyperalgesia (sensorimotor system). However, the existence of gender differences in schizophrenia has been described in humans^28,29^, therefore, future studies comparing male and female mice born to mothers administered LCM during pregnancy will give us information if this model also shows similar sex differences as humans.

At the cellular level, we also studied the effects exposing pregnant dams to LCM on the brain of their offspring. Histological analysis showed LCM provoked a dose-dependent increase in the relative size of the hippocampus and dentate gyrus, although the loss of doublecortin-labelled cells suggested adult hippocampal neurogenesis was impaired in the dentate gyrus of mice born to dams exposed to high doses of LCM. The hippocampus in general, and the dentate gyrus in particular, are brain areas associated with emotional and cognitive behaviours (revised in^30,31^). As adult hippocampal neurogenesis has been implicated in hippocampal activity, the alteration of neurogenesis of the adult hippocampus could affect learning and memory processes^32,33^, and/or behaviours related to anxiety and depressive symptoms^34,35^. We also found changes in neuron density in adult mice born to dams treated with high doses of LCM during gestation, mainly interneurons labelled with calretinin or calbindin in prefrontal cortex and amygdala respectively. These results suggest that the excitatory/inhibitory balance in these regions is distinct to that of healthy mice. Interestingly, the hippocampus, prefrontal cortex and amygdala are highly interconnected brain regions involved in sociability and fear behaviour^19,36–38^. Accordingly, these alterations may be associated with behavioural abnormalities related to schizophrenia spectrum disorders in adult male mice gestated in presence of LCM.

LCM may act by modulating the function of CRMP2 and/or VGSCs, both proteins that have been implicated in neurite outgrowth, axon/dendrite specification, neuronal polarity, progenitor proliferation and radial migration (for review^10,11^). Indeed, the effect of LCM on VGSCs may be mediated by CRMP-2^39^. Genetic mutation of CRMP-2 is embryonic-lethal (revised in^40^). Similarly, deletion of some VGSC subunits seems to alter the development of neuronal circuits, provoking foetal and postnatal lethality^41,42^. Exposure to high doses of LCM during gestation mimics the embryonic and early lethality found in mice carrying null mutations in CRMP-2 and VGSC subunit, although more mildly, suggesting that LCM may work through CRMP-2 and/or VGSCs.

Linkage studies and a linkage meta-analysis suggest an association between CRMP2 and schizophrenia in large family cohorts of various ethnic types^13,16,17^. Furthermore, functional genetic variants of CRMP2 have been associated with a risk of schizophrenia, with weaker CRMP2 expression in post-mortem brains of schizophrenia patients^12^. Indeed, mice carrying a conditional brain deletion for CRMP-2 develop behavioural alterations associated with schizophrenia^14,15^. The administration of high doses of LCM during gestation has a similar effect to the genetic inactivation of CRMP-2 in the brain, suggesting that a direct interaction of LCM with CRMP-2 may be responsible for both the teratogenic effect on mouse embryos and in adult male mice a phenotype associated with schizophrenia spectrum disorders. Future research should aim to clarify the mechanism by which LCM, by interfering with the activity of CRMP-2, increases the risk of developing embryopathies.

The presence of certain LCM metabolites in embryos, but not in the serum of the dams exposed to LCM, indicates the embryo actively metabolizes this compound distinctly. It is important to note that xenobiotic metabolism varies greatly from the embryo to the foetus, and throughout the animal´s life span^43,44^, meaning that enhanced or dampened activity of metabolizing enzymes could produce toxic metabolites of LCM that induce teratogenic effects. The intermediates formed by drug metabolism may be more water soluble than the original compound (parent) and thus, when formed within the embryo/foetus, they might not diffuse easily across membranes, and they may remain trapped inside the embryo, increasing the exposure time during development^45,46^. Moreover, the placenta does not protect the embryo from xenobiotic agents, since almost any drug in maternal circulation will cross the placenta^47^. Therefore, embryonic/foetal and placental drug metabolism could alter the pathway of LCM degradation, releasing teratogenic metabolites into the embryonic environment. However, much remains to be learned about embryo development and xenobiotic metabolism to fully evaluate the risks associated with exposure to drugs such as LCM *in utero*.

We reported here the possible increased risk of developing behaviour and cognitive alterations associated with schizophrenia spectrum disorders in off-spring born to mothers taking LCM. Classic genome sequencing studies produced evidence that mutations in genes associated with neurodevelopment are related to major neurological diseases like autism, epilepsy or schizophrenia^48^. However, new genome-wide studies are providing further insights into epigenomic changes in schizophrenia and also into the dynamic role of DNA methylation during early brain development. In this context, environmental risk factors can induce epigenetic changes that increase the risk of late schizophrenia^49^. Valproic acid is widely used as an AED, and it increases the risk of autism in rodents exposed prenatally to this drug. Moreover, these effects are not only limited to the first generation, but they also induce transgenerational inheritance of autism in a second and even third generation^50,51^. Additional studies focusing on the interaction of compounds like LCM in the epigenetic landscape of neurodevelopmental genes will help us to better understand neurological diseases.

Based on our data, administration of therapeutic doses of LCM to pregnant mice is not safe for the developing embryos and may cause growth retardation or major congenital malformations. Furthermore, such mice may be born with mild somatometric and reflexological defects, and in adulthood male mice may experience behavioural and morphological alterations associated with schizophrenia. On the basis of our data, it should be acknowledged that the risk of developing these effects is dose dependent when considering the administration of LCM to pregnant epileptic patients.

## Materials and methods

### Animals, LCM treatment and serum extraction and compliance with ethical standards

All procedures involving experimental animals were performed in compliance with local, national and European animal welfare laws, guidelines and policies. The approval of the Hospital Universitario Virgen del Rocío and the University Pablo de Olavide (Sevilla, Spain) animal care committees was obtained prior to performing this study in accordance with Spanish Royal Decree 53/2013, European Directive 2010/63/EU.

To assess the teratogenic potential of the drug for standard protocols, we used doses of LCM (10 mg/ml: Vimpat, UCB Pharma, Brussels, Belgic) that were multiples of the standard human doses^52^. For seizures, the recommended daily dose of LCM in humans is 200–600 mg/day^53,54^. Thus, we used 10, 40, 80 and 120 mg/kg in mice, which corresponded to 56.7, 227, 454 and 681 mg/day in humans, considering an average weight of human to be 70 kg and that of mice to be 30 gr^52,55^. Controls were administered the vehicle alone: saline solution 0.9% w/v. LCM was administered intraperitoneally in 0.4 ml of saline solution, given each day between 8:00 to 10:00 am. To reduce unnecessary stress to the animals, the LCM was administered once daily. A total of 32 dams were initially treated daily, two days before mating and until the 14^th^ day post-coitus. To investigate the effects of LCM on the postnatal and adult behavioural development of the offspring, 17 dams were treated two days post-coitus until birth. In the breastfeeding period, females were not under treatment given that a woman will bottle feed the baby if they were on medication.

Maternal weights were assessed at the end of the treatment periods. After 14.5 days of gestation (embryonic day14.5, E14.5), blood was extracted from the orbital sinus of the pregnant dams under anaesthesia (70 mg/kg Thiobarbital: Braun Medical S.A. Spain), as described previously^56^ and sacrificed by neck dislocation immediately after. Blood samples were centrifuged immediately, and the serum was collected and stored at -80 °C for further chemical analysis.

### Necropsy and histopathology

The E14.5 embryos were collected and fixed in 4% paraformaldehyde for 4 h and then kept at 4 °C in 70% ethanol. The litter size of each dam was recorded, and embryos were assessed for external malformations and photographed on a stereomicroscope (SteREO Discovery V8 + AxioCam Erc8, Zeiss, Oberkochen, Germany). The crown-rump lengths of embryos were measured in the photographs using ImageJ software (downloaded as a free software package from the public domain: http://rsb.info.nih.gov/ij/download.html) and based on the reference points described previously^57^. Embryos were then embedded in paraffin, sectioned at 10, 7 and 5 μm, and stained with Ehrlich Haematoxylin & Eosin (H&E) for histology analysis. Representative sections were selected from at least three embryos for each condition and photographed on an Olympus (Tokyo, Japan) BX-61 photomicroscope.

### General procedure for postnatal observations

In this part of the study, dams were treated until the day of delivery. After analysing the embryo toxicity data, the treatments were categorized as controls (saline vehicle solution alone), low dose (40 mg/kg) and high dose (120 mg/kg). All pregnant dams were allowed to deliver spontaneously and LCM administration was ceased on the day of delivery. The day of birth was designated as postnatal day (P) P0 and the litter size delivered by each dam was recorded, checking each pup for gross abnormalities. Pups were nursed by their natural dams until weaning and from PD 2, the pups were weighed and neonatal behavioural tests were carried out on 12 control mice, 12 low dose and 10 high dose mice from 7 litters. During testing whole litters were separated from the dams and maintained for 30 min in a warm environment. At this stage, males and females were pooled for neurodevelopmental screening. All testing was performed between 07:30 a.m. and 12:00 a.m., and the mice were weighed before performing the tests.

Neonatal behavioural tests were performed according to the protocols in the Fox battery^58^, with reflexology tests adapted as described previously^59^: In all reflex tests performed, the day the mice completed the reflex was reported.

#### Righting reflex

The animal was placed face up, and the time taken to turn over and adopt a prone position with all four feet on the ground was assessed. The maximum time allocated to perform the test was 30 seconds.

#### Cliff drop aversion

A mouse was placed at the edge of the table, with its forepaws and head extending over the edge. The response was scored as positive if the mouse turned around and crawled away at least 45° from the “cliff”. The maximum time allocated to perform this test was 30 seconds.

#### Negative geotaxis

The animal was placed face down on a 30° incline and the latency to turn 180° was recorded. The maximum time allocated to perform the test was 30 seconds.

#### Pivoting activity

The total number of degrees turned by the pup during a 30 s period was recorded. The test was performed on a flat surface covered with green paper on which lines had been drawn to define four 90° quadrants. The number of degrees was only scored in terms of completed 90° segments.

#### Walking test

The latency of a mouse to walk a distance exceeding its body length on all four legs was measured on a flat surface covered with green paper.

#### Suspension test

Mice were suspended by their forelimbs 20 cm above the work surface on a stretched cord about 20 cm long. The time taken to fall was measured up to a maximum of 1 minute.

#### Developmental landmarks

Pups were inspected daily for complete opening of both eyelids and separation of the ears. The day each landmark occurred was reported.

### Behavioural testing of adult mice

Three-month-old male mice exposed during gestation to the vehicle alone, or low and high doses of LCM, were evaluated in the behavioural tests^59^. 16, 17 and 19 mice (for vehicle, 40 and 120 m/kg LCM injected mice) from 10 litters were analysed for each behavioural test. All trials were performed by an experimenter blind to the drug treatment.

Behavioural tests included: Motor activity in the open field; Pain assay; Startle response/pre-pulse inhibition (PPI) test; Tail suspension test. Dark-light emergence task; Y maze; Sociability test; Predator test; and Object recognition memory. More detailed information can be found in the supplementary material.

### Nissl staining, immunohistochemistry and histological analysis

As formerly described by^59^, mice from each experimental group were anesthetized with 4% of chloral hydrate (10 µL/g of weight) and trans-cardiacally perfused with cold saline. Brains were removed and fixed by immersion for 24 h at 4 °C in 4% paraformaldehyde prepared in phosphate-buffered saline (PBS). Tissue was cryoprotected in 30% sucrose-PBS for 2 days at 4 °C and coronal brain sections (50 µm) were then processed for Nissl staining and free-floating immunohistochemistry. Sections encompassed roughly between 2.80 mm to -2.80 mm from Bregma^60^ were selected for Toluidine blue staining and immunohistochemical processing. Brain slices from all animals were processed simultaneously in each assay. Immunohistochemistry assays were performed with free-floating sections and an avidin-biotinylated peroxidase method. Primary antibodies used were calbindin (CB, 1:500, Santa Cruz, sc-365360), calretinin (CR, 1:1000, Santa Cruz, sc-26512) and doublecortin (DCX, 1:500, Santa Cruz, sc-8066). Secondary biotinylated antibodies were used (1:500). Signal was amplified by ABC kit reagents for 1 h (Vectastain PK-6100) and revealed with diaminobenzidine/nickel for colour deposition (SIGMAFAST DAB). Several control sections were processed without the primary antibody, showing no signal. To minimize variability, at least 2 sections from each area were analysed per animal on a bright-field DMRB RFY HC microscope (Leica) using a 10X objective. For DCX quantification images were taken using a 20X objective and later a whole image of dentate gyrus was reconstructed using Photoshop tiling function. In each section, the total number of labelled cells per area of tissue was quantified using Image-J software (downloaded as a free software package from the public domain: http://rsb.info.nih.gov/ij/download.html). 6 different animals were processed for each primary antibody and pharmacologic treatment from 10 litters.

### Analytical procedures to determine LCM and related compounds

#### Embryo and serum sample preparation

Samples were extracted as described previously^25^ with slight modifications. Briefly, embryos were rinsed with cold saline and ultrapure water, and their wet-weight was determined. They were then homogenized using a 150 T Ultrasonic homogenizer (Fisher Scientific, Spain) and 10 µL of an internal standard solution (IS: aqueous D^3^-LCM IS, 5 mg mL^−1^) was added to the biological samples (embryos or 50 µL of serum). Acetonitrile (300 µL) was then added to the mixture to precipitate the proteins, and to extract LCM and its related compounds. The mixture was vortexed vigorously for 3 min and centrifuged at 13,000 g for 5 min. The supernatant was transferred to a new Eppendorf tube and evaporated to dryness in a stream of nitrogen gas. The residue was reconstituted with 50 µL of LC-MS Ultrapure water, vortexed for 20 s and centrifuged at 13,000 g for 5 min. The supernatant was transferred to a conic micro insert to be injected onto the UHPLC system.

#### Analytical procedures for the determination of LCM and LCM and related compounds (RC), and possible metabolic routes in embryos

Detailed in supplementary material

### Statistical analysis

The results were analysed using the SPSS package for Windows and unless otherwise stated, the data are presented as the mean ± SEM values. The data were analysed with one-way ANOVA (for analysis of a parameter in the three pharmacological conditions), 2-way ANOVA (for analysis of several parameters in the three pharmacological conditions). After that, a t-test was used for post-hoc comparisons.

## Supplementary information

Supplementary information.
