# Supplementary material for: Lacosamide intake during pregnancy increases the incidence of foetal malformations and symptoms associated with schizophrenia in the offspring of mice
Source: Sci Rep. 2020 May 6;10:7615. doi: 10.1038/s41598-020-64626-9 (PMC7203245; doi:10.1038/s41598-020-64626-9)
Supplement: Supplementary file 1 — Supplementary information. [file 41598_2020_64626_MOESM1_ESM.docx]

**Lacosamide intake during pregnancy increases the incidence of foetal malformations and schizophrenia-like symptoms in the offspring of mice.**

**Beatriz López-Escobar^1*^, Rut Fernández-Torres^2,3*^, Viviana Vargas-López^4,5*^, Mercedes Villar-Navarro^2^, Tatyana Rybkina^4^, Eloy Rivas-Infante^6^, Ayleen Hernández-Viñas^1^, Concepción Álvarez del Vayo^7^, José Caro-Vega^1^, José A. Sánchez-Alcázar^8^, Antonio González-Meneses^9^, Ángel, M. Carrión^4¥^, Patricia Ybot-González^1,10,¥^**

**Supplementary material**

**Behavioural testing of adult mice**

Three month old male mice that had been exposed during gestation to the vehicle alone (n=16), or to low (n=17; 40mg/kg) or high doses of LCM n=19; 120mg/kg), were evaluated in the behavioural tests [^1^](#_ENREF_1) from 10 litters. All trials were performed by an experimenter blind to the drug treatment.

Behavioural tests were performed in a room with constant sound and light. To eliminate any odours or traces that might affect the outcome of the test, the behavioural apparatus was cleaned with 70% ethanol (Panreac Química S.A.U) between testing each animal. To avoid stress in our behavioural experiments the sequence in which the tests were performed was always the same always performing only one test per day: open field, object recognition memory, dark-light emergence test, and tail suspension (1st week); Y maze, plus maze, sociability, predatory test, and acoustic startle (2nd week); tail suspension, step down passive avoidance and hot plate test (3rd day). All mice performed the main part of the test (we performed two independent assays, but not the same tests were done in both of them).

***Motor activity in the open field.*** To evaluate locomotor and exploratory activity, mice were placed in an open field for 5 minutes (38 x 21 x 15 cm: Cybertec S.A., Madrid, Spain). This apparatus consisted of a walled platform containing infrared (IR) emitters and sensors coupled to an actimeter, and the movement sensor was connected to a computer that recorded the number of times the mouse interrupted the IR beams/min. Exploratory activity, was determined through the time the mice interacted with two different objects in a 15 minute session.

***Pain assay.*** Pain behaviour was assessed in mice using the hot plate. For the hot-plate test, a glass cylinder (16 cm high, 16 cm in diameter) was used to constrain the mice to the heated surface of the plate. The plate surface was maintained at 50-55 ± 0.5 ºC and the latency to commence paw-licking was measured, using a 30 s cut-off time for each of the tested temperatures. Each temperature was tested with 1h apart.

***Startle response/prepulse inhibition (PPI) test.*** Animals were placed individually inside a startle chamber (Cibertec S.A., Madrid, Spain) and the startle response was measured using a piezoelectric accelerometer controlled by a computer. The digitized signal was averaged from 25–30 recordings. For training, the mouse was placed in the startle chamber for an acclimation period of 3 min. Baseline responses were averaged after the presentation of 20 sounds (125 dB, 100 ms long) and from this phase, the average response and peak latencies, and the peak response were determined. During PPI trials, the same 125-dB 100-ms burst was preceded (250 ms) by a prepulse stimulus of 85 dB, lasting for 50 ms. Trials including prepulse stimuli were randomly presented with normal startle stimuli, the final total being 25 of each, and the proportion of PPI was determined as [(1-prepulse/startle)*100]. The ambient background noise was 70 dB.

***Tail suspension test.*** Depression-like behaviour in mice was determined by measuring the time spent immobile in the tail suspension test. Mice were suspended above the floor by attaching the end of their tail to wire netting, and the time spent immobile during a 5 min test session was measured.

***Dark-light emergence task.*** Anxiety-like behaviour was determined in mice using the dark-light emergence test. Testing was carried out in a white open field (50 x 50 cm) containing a small opaque chamber (12 x 12 cm) situated in the centre. The open field was illuminated by a lamp directed at the centre of the field (120 lux focused on the floor). Mice were moved to the testing room 1 hour prior to behavioural testing and they were introduced into the unfamiliar test environment by placing them into the small chamber and recording their behaviour over 5 minutes with a video camera. The total time spent outside the chamber was then quantified by reviewing the video recording.

***Y maze.*** The Y maze used had three equal sized arms (8 x 40 x 20 cm), with white opaque walls at a 120° angle from each other. After introduction to the centre of the maze, the mice was allowed to freely explore the three arms during 5 minutes, recording the number of arm entries and the number of triads in order to calculate the alternation. An entry was considered to occur when all four limbs are within the arm. The success of the triplet was taken into account when mice visited the three arms in three consecutive arm entries showing normal novelty preference and working memory.

***Sociability test.*** The social testing apparatus consisted of a rectangular arena (55 x 40 x 40 cm) in which a small, round wire cage (11 cm in height, with a bottom diameter of 9 cm, vertical bars 0.5 cm and horizontal bars spaced 1 cm apart) was placed in a corner, allowing nose contact between the bars but preventing fighting. The subject mouse was first placed in the arena for 5 minutes with the round wire cage empty. After that, a strange mouse was place in the round cage in the middle of the chamber and the subject was allowed to explore the entire social test box for 10 min. The time spent around the empty and occupied cage was measured with the aid of the camera fitted to the top of the box.

***Predator test.*** The predator test was performed in a rectangular arena (55 x 40 x 40 cm) where a small tube was placed in a corner. The subject mouse was first placed in the area with the empty tube for 5 mins and afterwards, the tube was filled with material from a cage that housed a rat and the mice were allowed to explore the entire fear test box for 10 mins. The time spent by the mice sniffing the tube full of the rat’s material was measured with the aid of the camera fitted to the top of the box.

***Object recognition memory.*** Mice were tested in a rectangular arena (55 x 40 x 40 cm) situated in a room with dim lighting and constant background noise. The object recognition protocol was described extensively elsewhere[^2^](#_ENREF_2). Briefly, two equal objects were placed in the arena during a 15 minutes training phase and the animal’s memory of the original object was assessed by comparing the amount of time spent exploring the novel object against that for the familiar one in two sessions (of 10 minutes) performed 1 and 24h after training (for short-term and long-term memories [STM and LTM] respectively). The time spent exploring each object was recorded and the relative exploration of the novel object was expressed by a discrimination index [DI = (t_novel_-t_familiar_)/(t_novel_+t_familiar_)]. The criteria for exploration were based strictly on active exploration. Exploration of an object was defined as directing the nose toward the object at a distance of 1.5 cm and/or touching the object with the nose or vibrissae. Circling or sitting on the object were not considered exploratory behaviours.

**Analytical procedures for the determination of LCM**

Biological samples were analysed by liquid chromatography coupled to quadrupole/time of flight mass spectrometry detection (UHPLC-QTOF/MS^e^), based on the previously reported methods [^3^](#_ENREF_3) with slight modifications. A Xevo G2-S QTOF mass spectrometer (Waters, Micromass, Manchester, UK) coupled to an Acquity ultra performance liquid chromatography (UPLC) system (Waters, Micromass, Manchester, UK) was used with an orthogonal Z-spray lockspray and electrospray interface. The chromatographic separations were achieved on an Acquity BEH C18 analytical column (50 mm x 2.1 mm i.d., 1.7 µm particle size: Waters, Micromass, Manchester, UK). For MS and MS/MS determination, isocratic elution was carried out with a mobile phase consisting of water (85%) and acetonitrile (15%) at a flow rate of 0.5 mL/min. The injection volume was 5 µL and the analysis time was 5 minutes. For MS^e^ determinations, a gradient elution program was employed using a mobile phase that consisted of water (A) and acetonitrile (B). The proportion of B increased linearly from 10% at the outset to 90% after 10 minutes, at a flow rate of 0.5 mL/min, and returning to the initial value for two minutes prior to a new injection. The injection volume was 5 µL, and the column and autosampler tray were maintained at 25 °C and 5 °C, respectively.

The column was eluted into the mass spectrometer through the turbo ionspray, operating in a positive mode to detect LCM at m/z 251.139 and an internal standard at 255.161, with typical settings as follows: cone gas 0 L/h (nitrogen); desolvation gas 960 L/h (nitrogen); cone voltage 31 V; capillary voltage, 1.5 kV; desolvation temperature 400 ºC and source temperature 120 ºC. The molecular ions of LCM were fragmented at a collision energy of 10 eV with a cone voltage 10 V in the collision cell with argon gas (99.995%, Praxair). MS spectra data were collected between m/z 50 and 1000, and MS/MS spectra were registered between 50-300 Da. The detection of the LCM ion was confirmed by monitoring the transition of m/z 251.139 to fragment ions 91.052, 108.081, 219.112 in the MS/MS spectra. The MS^e^ procedure was used to identify metabolites in positive ionization mode. UPLC–QTOF-MS^e^ detection collects data using two scan functions, providing fragment ion information without precursor ion selection. A characteristic *m/z* in the low energy function allows identification through the fragment ions obtained with the high collision energy function. Two functions were acquired at different collision energies: a low energy function obtained at 2 eV; and a high-energy function obtained using a collision energy ramp ranging from 15 to 30 eV and spectra data collected between m/z 50 and 800. The data station operating the software was a MassLynx version 4.1, while metabolites were identified by means of Metabolynx XS (Waters, Micromass, Manchester, UK). The analytical measurements were in the 0.5 to 500 ng/mL range (calibration graphs were constructed based on the peak area ratio LCM/IS vs LCM concentration). Intra-assay and inter-assay precision were 2.9 % and 8.7% (relative standard deviation) respectively at 10 ng/mL. The detection limit for LCM in the plasma and embryos was 0.5 ng/mL, and the coefficient of variation of the assay (within and between days) were generally low (< 8.9%). Recovery assays were realized on placebo samples spiking at 10 ng/mL (n=10), in all cases obtaining recoveries of 85 ± 5 %.

**LCM and related compounds (RC), and possible metabolic routes in embryos**

The presence of LCM related compounds was investigated by target analysis of the MS^e^ data using MetaboLynx software (Waters, Micromass, Manchester, UK). This software compares results from samples with the control samples (placebos), reporting ions and chromatographic signals that match possible metabolites corresponding to the main metabolic routes of interest previously defined by the user. In this work the following routes were selected for these searches: phase I metabolites Reduction (+H_2_), Hydroxylation (O), Hydration (+H_2_O), 2xHydroxylation (+O_2_), Deacetylation (-C_2_H_2_O) and Demethylation (-CH_2_); and phase II metabolites Acetylation (+C_2_H_2_O) and Glucuronide (+C_6_H_8_O_6_). From the accurate mass obtained, the software calculates the nature of the possible metabolites according to their structure and the composition of the original LCM.

Two degradation products described previously [^3^](#_ENREF_3) that corresponded to the protonated benzyl amine of LCM (RC-1, *m/z* 108.0802, C_7_H_9_N) and O-Demethyl LCM (RC-2, *m/z* 237.1222, C_12_H_16_N_2_O_3_) were detected in 1 embryo from a dam treated with 10 mg/kg LCM (RC-1), and in 2 embryos from dams treated with 10 mg/kg and 80 mg/kg LCM (RC-2). In addition, a number of related compounds, not previously reported, were also identified in some samples. RC-3 (m/z 104.0727, C_4_H_9_NO_2_) was found in 2 embryos of mothers who received 10 mg/kg and 40 mg/kg LCM. Its molecular formula suggests a first deacetylation of LCM followed by the loss of a C_7_H_7_N fragment. Our results also showed that the samples from the mothers who received 10 mg/kg LCM contained deacetylacosamide (m/z 209,1289), suggesting that in a first step the LCM undergoes a deacetylation in the mother and then, the loss of a C_7_H_7_N fragment in the embryo to obtain RC-3. RC-4 (*m/z* 135.0797, C_9_H_10_O) was found in 2 embryos from a dam that received 40 mg/kg LCM, and this compound matches with a first acetylation of LCM and then the loss of C_6_H_10_N_2_O_3_; this compound was also found in the corresponding dams samples. RC-5 (*m/z* 217.1062, C_11_H_15_NaO_2_) was found in 3 embryos from dams receiving 10, 40 and 80 mg/kg LCM and its molecular formula suggests the loss of C_2_H_3_NO in the LCM sodium adduct. RC-6 (*m/z* 236.1278, C_13_H_17_NO_3_) and RC-7 (*m/z* 284.1145, C_13_H_17_NO_6_) were found together in 2 embryos from dams that received 40 and 80 mg/kg LCM, RC-6 alone was identified in an embryo born to a dam that was administered 80 mg/kg LCM, and RC-7 in an embryo from a litter exposed to 40 mg/kg LCM. The formula of RC-6 suggests an initial acetylation of LCM and then the loss of C_2_H_3_NO. Interestingly, dams that received 40 and 80 mg/kg showed metabolites derived from the metabolic pathways of acetylation. However, none of the maternal samples contained the acetylacosamide metabolite found in the embryos, but rather related compounds that undergo acetylation in a first step and then the loss of different fragments. RC-7 would correspond to a second phase metabolite where LCM suffers a glucuronide conjugation. RC-8 (m/z 180.1022 C_10_H_13_NO_2_), RC-9 (m/z 211.1437, C_11_H_18_N_2_O_2_) and RC-10 (m/z 283.1276, C_13_H_18_N_2_O_5_) were found in the 2 embryos analysed from dams that received 120 mg/kg LCM. RC-8 matches to the demethylation of LCM and then the loss of C_2_H_3_NO. We found that some samples from the corresponding dams contained desmethylacosamide which suggests a first step of metabolism in the mother and then this compound would undergo a second transformation through the metabolism of the placenta or the embryo. The molecular formula of RC-9 suggests an initial reduction of LCM and then the loss of C_2_H_2_O, while RC-10 appears to proceed from the double hydroxylation of LCM. These two RCs were neither found in dam samples nor their related precursors.

1. Romero-Granados, R., Fontan-Lozano, A., Aguilar-Montilla, F. J. & Carrion, A. M. Postnatal proteasome inhibition induces neurodegeneration and cognitive deficiencies in adult mice: a new model of neurodevelopment syndrome. *PLoS One*. **6**, e28927 (2011).

2. Suarez-Pereira, I., Canals, S. & Carrion, A. M. Adult newborn neurons are involved in learning acquisition and long-term memory formation: the distinct demands on temporal neurogenesis of different cognitive tasks. *Hippocampus*. **25**, 51-61 (2015).

3. Ramisetti, N. R., Kuntamukkala, R., Lakshetti, S. & Sripadi, P. Identification and characterization of stress degradants of lacosamide by LC-MS and ESI-Q-TOF-MS/MS: development and validation of a stability indicating RP-HPLC method. *J Pharm Biomed Anal*. **95**, 256-264 (2014).
